# Supplementary material for: Predation and fragmentation portrayed in the statistical structure of prey time series
Source: BMC Ecol. 2009 May 6;9:10. doi: 10.1186/1472-6785-9-10 (PMC2689204; doi:10.1186/1472-6785-9-10)
Supplement: Additional file 2 — Voles and related classes ODDox Documentation. ODDox documentation of the agent-based model (ALMaSS) applied by Hendrichsen et al. The documentation is started by activating main.html. [file 1472-6785-9-10-S2.zip › Vole_ODDox/_vole_population_manager_8_h.html]

ALMaSS ODDox: VolePopulationManager.H File Reference

- Main Page
- Related Pages
- Classes
- Files

# VolePopulationManager.H File Reference

---

## Detailed Description

**VolePopulationManager.h This is the header file for the vole population manager class**   

by Chris J. Topping   
Version of 28th Jan 2001   
All rights reserved.   
  
With additions as noted in:   
April 2006   
November 2007   
Doxygen formatted comments in May 2008   

|  |  |
| --- | --- |
|  |  |
| Classes | |
| class | Vole\_Population\_Manager |
|  | The class to handle all vole population related matters. More... |
| Typedefs | |
| typedef vector< Vole\_Female \* > | Vole\_FemaleList |
| typedef vector< Vole\_Male \* > | Vole\_MaleList |
| Enumerations | |
| enum | TTypeOfVoleMessage { tovm\_Infanticide = 0 } |
|  | Types of vole message. More... |

---

## Typedef Documentation

typedef vector<Vole\_Female \*> Vole\_FemaleList

typedef vector<Vole\_Male \*> Vole\_MaleList

---

## Enumeration Type Documentation

enum TTypeOfVoleMessage

Types of vole message.

**Enumerator:**
:   |  |  |
    | --- | --- |
    | *tovm\_Infanticide* |  |

```
00041               {
00042         tovm_Infanticide=0
00043 }TTypeOfVoleMessage;
```

---

Generated on Thu Jan 22 14:13:45 2009 for ALMaSS ODDox by 
 1.5.6
